# Establishing Reference Intervals for White Blood Cell and Absolute Neutrophil Counts in Duffy Null Individuals

**DOI:** 10.3390/hematolrep18030038

**Published:** 2026-06-08

**Authors:** Mohammad Barouqa, Muhammad Areeb Ashfaq, Katrina J. Jiang, Huseyin Kilic, Nestor Dela Cruz, Aariez Khalid, Marianne E. Yassa

**Affiliations:** 1 Department of Pathology, University of South Alabama, 2451 University Hospital Dr, Mobile, AL 36617, USA; 2 Department of Internal Medicine, Hematology and Medical Oncology Division, University of South Alabama, 2451 University Hospital Dr, Mobile, AL 36617, USA; 3 Department of Internal Medicine, University of South Alabama, 2451 University Hospital Dr, Mobile, AL 36617, USA

**Keywords:** Duffy-null, neutropenia, leukopenia, absolute neutrophilic count, white blood cell count

## Abstract

**Background/Objectives:** The Duffy (Fy) blood group system, encoded by the *ACKR1* gene, plays a key role in transfusion medicine and host susceptibility to malarial infections such as *Plasmodium vivax*. The Duffy-null phenotype [Fy(a−b−)] is associated with lower baseline white blood cell (WBC) and absolute neutrophil count (ANC) values despite a benign clinical course. This study aimed to establish specific reference intervals for WBC and ANC in Duffy-null individuals to improve diagnostic accuracy and reduce unnecessary clinical interventions. **Methods:** We conducted a retrospective analysis of 1695 adult patients who underwent complete blood count testing and red blood cell phenotyping between 1 January 2020 and 1 January 2026. Among these, 122 healthy individuals with confirmed Fy(a−b−) phenotype met inclusion criteria. Reference intervals were established using 95% reference interval nonparametric methods in accordance with Clinical Laboratory Standards Institute (CLSI) guidelines. **Results:** The median WBC and ANC values in the Duffy-null cohort were significantly lower than institutional reference medians (3.24 vs. 6.65 × 10^3^/µL and 1.45 vs. 4.45 × 10^3^/µL, respectively; *p* < 0.01). The derived 95% reference intervals (WBC: 2.43–7.05 × 10^3^/µL; ANC: 1.01–4.34 × 10^3^/µL) fell below conventional thresholds, with the lower ANC bound below the standard neutropenia cutoff. **Conclusions:** These findings support the need for Duffy-null-specific hematologic reference ranges. Adoption of such intervals may reduce misclassification, avoid unnecessary diagnostic procedures, and promote personalized clinical care.

## 1. Introduction

Red blood cells (RBCs) express different groups of antigens on their surfaces which characterize them into different groups. These polymorphic antigens can vary in sequence and structure and are classified into groups which include ABO system, Rhesus group, Kell group and Duffy group with a total of 47 blood groups systems recognized comprising 366 antigens per the International Society of Blood Transfusion (ISBT) [1,2]. The Duffy (Fy) antigen, first characterized in 1951, is a transmembrane chemokine receptor expressed in many tissues including RBCs, Purkinje cells in the cerebellum, lung alveoli, capillary endothelial cells, airway smooth muscles, thyroid, spleen and cochlear hair cells [3,4,5]. Duffy antigens are expressed by the *ACKR1* gene located on chromosome 1q23.2, and mainly there are two key antigens in this group, Duffy-a (Fy(a)) and Duffy-b (Fy(b)). Both antigens are encoded by co-dominant FY*A(FY*01) and FY*B(FY*02) alleles, differentiated by a single base amino acid (Gly42Asp, which categorize phenotypes into Fy(a+b−), Fy(a−b+) and Fy(a+b+)) [1,6]. Duffy antigens are also associated with malarial infections, specifically Plasmodium vivax and Plasmodium knowlesi, where the protozoa requires the presence of Fy(a) or Fy(b) expression on the surface of RBCs for infection [7]. The Duffy blood group system and its associated alloantibodies are of considerable importance in transfusion medicine because of their potential to cause hemolytic transfusion reactions and hemolytic disease of the fetus and newborn (HDFN), as well as their implicated roles in renal transplantation and graft rejection [8].

The Duffy-null phenotype (Fy[a−b−]) is caused by the ACKR1 rs2814778 promoter variant and shows variable global allele frequencies. Higher prevalence has been observed with historical exposure to malaria-endemic regions, specifically Plasmodium vivax. [9]. The null phenotype is the result of a single nucleotide variant in the GATA-1 transcription factor binding site located in the *ACKR1* promoter gene (*rs2814778*) that results in preventing the expression of the Fy(a) and Fy(b) genes and their associated glycoproteins. This alteration is thought to influence hematopoiesis, resulting in a distinct neutrophil profile characterized by an increased propensity for migration from the peripheral circulation into tissues [10].

The Duffy-null phenotype has been increasingly associated with lower but clinically benign peripheral white blood cell and absolute neutrophil count [11], mainly detected incidentally in patients during routine visits to clinics and commonly referred to hematology clinics for follow up [9,12]. These patients are characterized by lower white blood cell count (approximately 700 cells per μL lower) and consequently lower absolute neutrophil count with a benign clinical course and no disease susceptibility [9]. Despite its benign clinical course, this finding has been associated with unnecessary diagnostic interventions, including bone marrow biopsies, as well as unwarranted enrollment in clinical trials and initiation of treatments [13,14]. Hibbs et al. estimated that the use of institutional reference intervals resulted in the misclassification of individuals as neutropenic, affecting 21.7% in the United States, 26.0% in the United Kingdom, 50.9% in Saudi Arabia and 27.9% of participants in Namibia concluding that current reference ranges overlook this benign variation [15].

Given that an estimated 70% of clinical decisions rely on laboratory test results, the use of hematologic reference intervals derived from the general healthy populations may disproportionately impact Duffy-null individuals, resulting in misclassification and potentially leading to unnecessary diagnostic procedures, referrals, and iatrogenic harm [11]. Although WBC reference intervals for Duffy-null individuals have been recently published by several institutions, they remain less extensively studied. Hence, we sought to analyze and establish reference intervals for white blood cell (WBC) count and absolute neutrophil count (ANC) specifically for Duffy-null patients with the goal of improving diagnostic accuracy and reducing potential misclassification and unnecessary clinical interventions in this group.

## 2. Materials and Methods

Using the laboratory information system (LIS; PathNet, Cerner Build 1.1.1.1, Kansas City, MO, USA), we identified 1695 adult (age ≥ 18 years old) patients who underwent complete blood count (CBC) testing and red blood cell (RBC) phenotyping at the University of South Alabama Medical Center in Mobile, Alabama, between 1 January 2020 and 1 January 2026. From this cohort, individuals with the Fy(a−b−) phenotype were identified (*n* = 610). A retrospective chart review was then performed on this subset to determine eligibility based on predefined exclusion criteria, including autoimmune disease, malignancy, active pregnancy, organ transplantation, and use of medications known to affect leukocyte counts. After application of these criteria, 122 individuals met inclusion criteria and were classified as healthy Duffy-null participants for analysis.

CBC was performed on the Sysmex XN-9100 analyzer (Sysmex, Kobe, Japan), and RBC phenotyping was performed manually in tube using monoclonal anti-Fy(a) and anti- Fy(b) reagents (Immucor, Norcross, GA, USA). EP evaluator (V.12.3.0.2) was used to establish a 95% reference interval by nonparametric percentile method (2.5th and 97.5 percentiles) in accordance with CLSI guidelines [16]. RBC phenotyping and CBC testing were performed within the same clinical encounter. R-Studio (V.2024.9.0) was used to perform statistical analysis including a 1-sample, 1-sided Wilcoxon signed rank test to compare the WBC and ANC medians established in the laboratory’s reference interval with the Duffy-null cohort median with a *p*-value < 0.05 deemed statistically significant.

## 3. Results

A total of 122 individuals were included in this cohort for the establishment of white blood cell (WBC) and absolute neutrophil count (ANC) reference intervals. All patients were serologically phenotyped as Fy(a)- and Fy(b)-negative, and none had any history of previous transfusion within 90 days of serologic phenotyping. Forty-seven out of 122 (38.5%) had historical molecular phenotyping that detected thers2814778 in the GATA1 promoter gene of Fy(b) gene. The mean age was 37.8 years, with a median age of 36.5 years, indicating a relatively young cohort with a concentration of participants in middle adulthood. The cohort consisted primarily of African American individuals (119/122, 97.5%), with a proportion classified as Other (3/122, 2.5%). Most participants were non-Hispanic (121/122, 99.2%), with one participant identifying as Hispanic (0.8%). Furthermore, it included 73 females (59.8%) and 49 males (40.2%). Race and ethnicity are reported for descriptive epidemiology only and not used as biological variables in this analysis.

In the Duffy-null cohort, the median white blood cell (WBC) count was 3.24 × 10^3^/µL (interquartile range [IQR], 3.02–3.84 × 10^3^/µL; range, 2.29–9.2 × 10^3^/µL). The median absolute neutrophil count (ANC) was 1.45 × 10^3^/µL (IQR, 1.26–1.76 × 10^3^/µL; range, 0.81– 5.23 × 10^3^/µL).

Median WBC and ANC values in the Duffy-null cohort were significantly lower than the laboratory-established reference medians (3.24 vs. 6.65 × 10^3^/µL for WBC and 1.45 vs. 4.45 × 10^3^/µL for ANC; *p* < 0.01 for both comparisons).

**Figure 1:**
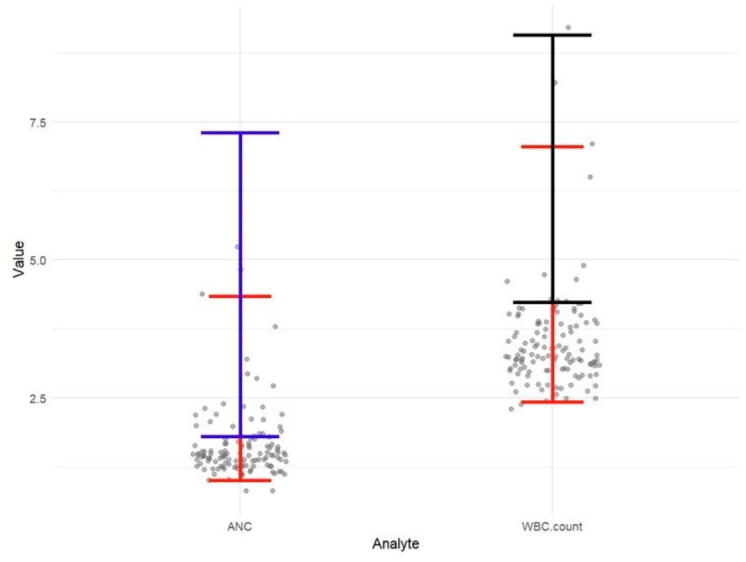
Comparison of reference intervals between the Duffy-null cohort and laboratory-established ranges. The red line represents the Duffy-null cohort. Blue and black lines represent the laboratory-established reference intervals.

The laboratory reference interval for WBC (4.23–9.07 × 10^3^/µL) was higher than the nonparametric central 95% interval observed in the Duffy-null cohort, which ranged from 2.43 × 10^3^/µL (90% CI, 2.29–2.61 × 10^3^/µL) to 7.05 × 10^3^/µL (90% CI, 4.64–9.20 × 10^3^/µL). Similarly, the laboratory reference interval for ANC (1.80–7.30 × 10^3^/µL) exceeded the Duffy-null central 95% interval, which ranged from 1.01× 10^3^/µL (90% CI, 0.81–1.11 × 10^3^/µL) to 4.34 × 10^3^/µL (90% CI, 2.85–5.23 × 10^3^/µL) (Figure 1).

## 4. Discussion

This study establishes a specific reference interval for white blood cell (WBC) and absolute neutrophil count (ANC) in individuals with the Duffy-null [Fy(a−b−)] phenotype and reinforces a growing body of evidence that conventional hematologic reference intervals should be refined in clinical laboratories. Our findings indicate that both WBC and ANC values in Duffy-null individuals are significantly lower than conventional laboratory reference medians yet remain physiologically normal and clinically benign. These results are in consensus with previous studies and highlight the need for greater consideration of Duffy-null status in diagnostic stewardship and clinical decision making [15,17].

The statistically significant difference between our cohort and institutional reference medians (*p* < 0.01) highlights the limitations of applying generalized reference intervals derived from the general population. Importantly, the lower bound of the 95% reference interval for ANC (1.01 × 10^3^/µL) falls well below the conventional clinical threshold for neutropenia (1.5 × 10^3^/µL), suggesting that a substantial proportion of healthy Duffy-null individuals would be misclassified as neutropenic using current standards which may trigger further unnecessary testing and treatments.

On the molecular level, these findings align with the known effects of the rs2814778 polymorphism in the ACKR1 gene promoter, which abolishes erythroid expression of Duffy antigens while preserving expression in non-erythroid tissues. This alteration has been associated with enhanced neutrophil egress from the peripheral circulation into tissues rather than impaired production, thereby explaining the lower circulating neutrophil counts without an increased risk of infection. Our data support this model, as all included individuals were clinically healthy and lacked evidence of recurrent infections or hematologic pathology.

From a clinical perspective, the implications of these findings are substantial. The reliance on standardized laboratory thresholds, estimated to inform approximately 70% of medical decisions, can lead to unnecessary diagnostic evaluations in Duffy-null individuals, including repeated laboratory testing, hematology referrals, and invasive procedures such as bone marrow biopsy. Furthermore, misclassification of neutropenia may result in inappropriate exclusion from clinical trials or unwarranted dose modifications of myelosuppressive therapies, particularly in oncology and immunosuppressive treatment settings. By establishing tailored reference intervals, our study provides a framework to mitigate these risks and promote more accurate interpretation of laboratory data.

To integrate these findings into clinical practice, clinical laboratories may consider implementing Duffy-null-specific reference intervals within laboratory information systems for individuals with confirmed Fy(a−b−) phenotype or ACKR1 rs2814778 genotype. Practical application may include parallel reporting of adjusted WBC and ANC reference ranges alongside conventional intervals, supported by standardized interpretive comments in laboratory reports. In settings where routine phenotyping or genotyping is not available, reflex testing strategies and clinical flags for persistent, isolated low neutrophil counts in otherwise asymptomatic patients may help identify individuals and notify care providers to consider this null phenotype in their differential diagnosis. It is crucial that laboratory approach and clinical interpretation remain explicitly genotype- and/or phenotype-based to avoid extrapolation beyond the biologic mechanism.

The limitations of our study include the retrospective design which introduces potential selection bias, and although rigorous exclusion criteria were applied, unrecognized confounders may have influenced hematologic parameters. Additionally, the sample size, while meeting Clinical Laboratory Standards Institute (CLSI) recommendations for reference interval determination, remains modest. Finally, we did not evaluate longitudinal trends or clinical outcomes, which could further validate the benign nature of these hematologic profiles over time. Furthermore, the potential impact of misclassification under conventional reference intervals could not be directly quantified in this study. Despite these limitations, this study provides data supporting the adoption of Duffy-null-specific reference intervals. Implementation of such ranges in laboratory reporting systems, guided by RBC phenotyping or genotyping, could significantly reduce unnecessary interventions and improve patient care. Moreover, these findings highlight a broader need to incorporate serologic and genetic phenotyping into laboratory medicine, moving toward a more accurate approach to diagnostics.

## 5. Conclusions

In conclusion, individuals with the Duffy-null phenotype exhibit significantly lower WBC and ANC values compared to conventional reference intervals, without associated clinical pathology. Recognition and integration of these differences into routine clinical practice and laboratories reviews are essential to prevent misdiagnosis, avoid unnecessary procedures and enhance the precision of medical care.

## Data Availability

The data presented in this study are available on request from the corresponding author and are not publicly available due to privacy or ethical restrictions.
